# Association of cervical microbial community with persistence, clearance and negativity of Human Papillomavirus in Korean women: a longitudinal study

**DOI:** 10.1038/s41598-018-33750-y

**Published:** 2018-10-19

**Authors:** Selvaraj Arokiyaraj, Sang Soo Seo, Minji Kwon, Jae Kwan Lee, Mi Kyung Kim

**Affiliations:** 1 Division of Cancer Epidemiology and Management, Center for Uterine Cancer, National Cancer Center, Ilsandong-gu, Goyang, Republic of Korea; 2 Department of Food Science and Biotechnology, Sejong University, Gwangjin-gu, Seoul, Republic of Korea; 3 Department of Obstetrics and Gynecology, Korea University College of Medicine, Seoul, Republic of Korea

## Abstract

The present study aimed to identify the cervical microbes that are associated with HPV negativity, HPV clearance and HPV persistence and to assess the microbes’ longitudinal associations as related to HPV infection dynamics among Korean women. We enrolled 41 women with 107 samples, and classified them according to the HPV infection dynamics: HPV negativity (21 samples, 10 subjects), HPV clearance (42 samples, 15 subjects), and HPV persistence (44 samples, 16 subjects). Cervical swabs were collected at the baseline and six-month-interval follow-up visits. HPV positivity was determined by HPV DNA HC2 assay, and the microbiome was analyzed using 16SrRNA pyrosequencing, linear discriminant analysis effect size and multivariate logistic analysis. In the multivariate logistic analysis results, *Lactobacillus crispatus* (multivariate OR (mOR) = 8.25, 95% CI 2.13~32.0) was predominant in the HPV-negative group. We observed that *Eubacterium eligens* (mOR = 11.5, 95% CI 1.31~101.4), *Gardnerella vaginalis* (mOR = 17.0, 95% CI 2.18–131.8), and *Ureaplasma urealyticum* (mOR = 7.42, 95% CI 1.3–42.46) had the strongest associations with HPV clearance, and *Lactobacillus johnsonii* (mOR = 16.4, 95% CI 1.77–152.2) with HPV persistence. Overall, greater diversity was observed in HPV-persistence than in HPV-negative women. Our findings suggest that the presence and prevalence of a specific cervical microbiome are factors involved in HPV dynamics.

## Introduction

Cervical cancer is one of the leading causes of cancer death among women, and the most important causal agent in the development of cervical intraepithelial neoplasia (CIN) and cervical cancer is persistent infection with high-risk human papilloma virus (hrHPV)^1,2,4,5^. The typical pattern of progression in HPV-mediated cervical cancer is as follows: (i) acquisition, (ii) persistence, (iii) progression to pre-cancer (CIN 1, 2 and 3), and invasive cancer. There is evidence that most cases of HPV infection are transient, which is to say, likely to regress naturally^3^. Several cofactors such as smoking, high parity, long-term use of oral contraceptives^6^, hormone treatment and co-infection with sexually transmitted infection agents are relevant to progression of cervical cancer among HPV-infected women^7^. Associations between cervical microbes and HPV infection and CIN have been investigated^8,9^. Also, there have been reports on, for example, the frequency and determinants of acquisition and persistence of HPV infection among Danish soldiers^10^ and the viral and non-viral determinants of cervical HPV acquisition and clearance in Hawaiian women^11^. Recently too, an American study reported temporal changes of cervical microbes and HPV detection among Baltimore women^12^. Notwithstanding the many reports on cervical microbes and their HPV associations^13,14^, more knowledge on the epidemiology and longitudinal dynamics of cervical microbes is required before the nature of HPV progression is clearly understood. The present study aimed to identify the cervical microbes that are associated with HPV negativity, HPV clearance and HPV persistence and to assess the microbes’ longitudinal associations as related with HPV infection dynamics among Korean women.

## Results

### General characteristics

We enrolled 41 women and classified them into three HPV groups according to the HPV infection dynamics: HPV negativity (21 samples, 10 subjects), HPV clearance (42 samples, 15 subjects), and HPV persistence (44 samples, 16 subjects). The epidemiological and clinical information on the subjects is presented in Table 1. There were no significant differences in the age at enrollment among the HPV groups. Also, the HPV viral load at enrollment did not differ between HPV clearance and HPV persistence.

**Table 1:** Demographic characteristics of study participants at baseline among HPV groups (Negative, Clearance, and Persistence).

Demographic characteristics	Negative (N = 10) 21 sample	Clearance (N = 15) 42 sample	Persistence (N = 16) 44 sample	*p* value
Age (years)	44.6 ± 9.0	44.4 ± 13.3	44.6 ± 13.0	0.982
HPV, viral load); Median (range)	—	34.6 (1.13–97.7)	37.4 (2.67–1466.8)	0.603
Body mass index (Kg/m^2^)	22.1 ± 1.9	22.9 ± 3.4	21.8 ± 3.5	0.592
Parity(parous)	2.38 ± 0.5	2.27 ± 0.6	2.42 ± 1.4	0.311
Postmenopausal status	2 (20)	7 (50)	6 (37.5)	0.326
Oral contraceptive use (ever)	3 (30)	5 (33)	1(6)	0.157
HRT				
*Never*	9 (90)	13(87)	13(81)	1
*Ever*	1 (10)	2(13)	3(19)	
Smoking status				
*Never*	8 (80)	12(80)	15(94)	0.537
*Ever*	2 (20)	3(20)	1(6)	
Passive smoking				
*No*	6 (60)	8(57)	11(69)	0.792
*yes*	4 (40)	6(43)	5(31)	
Alcohol drinking status				
*Yes*	8 (80)	11(79)	12(75)	1.0
*No*	2 (20)	3(21)	4(25)	
Alcohol drinking (>1 week)	1 (10)	5 (36)	5 (31)	0.480

Values are Mean ± SD or n (%). Only available variables were used in this study, due to missing response for several questions. Chi square test was used for testing categorical variables and Kruskal Wallis test for comparisons of continuous variables. When the number of expected frequencies was less than 5 and the number of cells is more than 25%, Fisher’s exact test was performed. HRT: Hormone replacement therapy; HPV: human papillomavirus.

### Cervical microbiota among HPV group

We sequenced 107 cervical samples (41 subjects) from a total of 617, 044 high-quality reads with an average of 18.97 operational taxonomic units (OTUs) per sample after quality filtering. The sequence reads were assigned to 14.5 OTUs in HPV negativity, 20.2 OTUs in HPV clearance, and 24.5 OTUs in HPV persistence. Eleven phyla — Firmicutes, Actinobacteria, Bacteroidetes, Fusobacteria, Proteobacteria, Tenericutes, Cyanobacteria, Verrucomicrobia, Planctomycetes, Acidobacteria, and Candidatus Saccharibacteria — were found, six of which (Firmicutes, Actinobacteria, Bacteroidetes, Fusobacteria, Proteobacteria, and Tenericutes) were dominant (>1%) among the groups. *Lactobacillus crispatus* and *Lactobacillus iners* were the dominant species among the three HPV groups. A shift in the cervical microbiota was observed during the follow-up period (Supplementary Table S1). The HPV-negative women showed the highest abundance of *Lactobacillus crispatus* at the baseline (40.45%) and six-month-interval follow-up visits (35.6%). Compared with HPV-negative, HPV positive-women (HPV clearance + persistence group at baseline) had the highest abundance of *Atopobium vaginae* at the baseline and follow-up visits. Compared with HPV persistence, HPV clearance women showed increased percentage of *Eubacterium eligens*, *Ureaplasma urealyticum* at the baseline and follow-up visits. HPV persistence women showed high proportion of *Lactobacillus johnsonii* at the baseline and follow-up visits, compared with other groups (HPV negativity + clearance group).

### Identification of cervical microbiome markers of HPV dynamics

**Figure 1:**
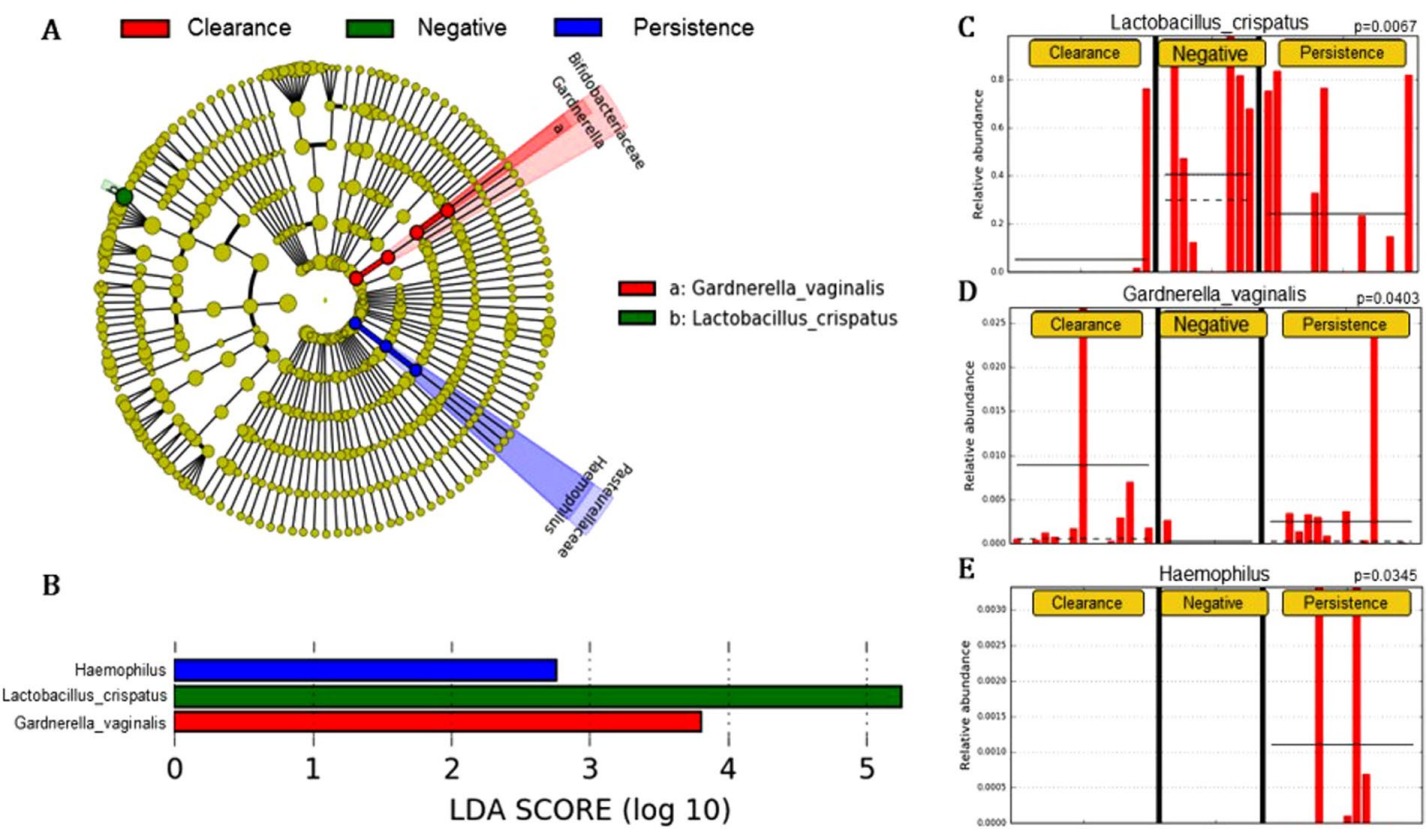
(**A**) Cladogram representing taxa with different abundances according to HPV groups at baseline visit. Red and green colors show taxa enriched in clearance and persistence women. Brightness is proportional to the abundance of taxa. (**B**) Histogram of Linear Discriminant Analysis (LDA) scores computed for features differentially abundant among HPV groups (clearance, negative and persistence). The LDA score on the log_10_ scale is indicated at the bottom. The greater the LDA score is, the more significant the phylotype biomarker is in the comparison. Relative abundance counts of *Lactobacillus crispatus, Gardnerella vaginalis* and *Haemophilus* which were found to be significantly over-represented in the HPV negative (**C**) HPV clearance (**D**) and HPV persistence (**E**) groups.

The taxonomic groups that were relatively abundant in the HPV persistence, negativity and clearance groups were identified by linear discriminant analysis effect size (LEfSe with α = 0.05), an LDA score of at least 2, and a relative abundance greater than 0.1, both at the baseline and for total visits. The changes at the baseline are depicted in a cladogram (Fig. 1A). In the HPV-persistence group, significant over-representation of Haemophilus (Order: Pasteurellales; Family: Pasteurellaceae) (P = 0.0345) (Fig. 1A,B,E) was observed. By contrast, the HPV-clearance group was found to have significantly higher levels of *Gardnerella vaginalis* (Order: Bifidobacterialies; Family: Bifidobacteriaceae) (P = 0.0403) (Fig. 1A,B,D), and the HPV-negative group was determined to have significant overrepresentation of *Lactobacillus crispatus* (P = 0.0067) (Fig. 1A–C).

**Figure 2:**
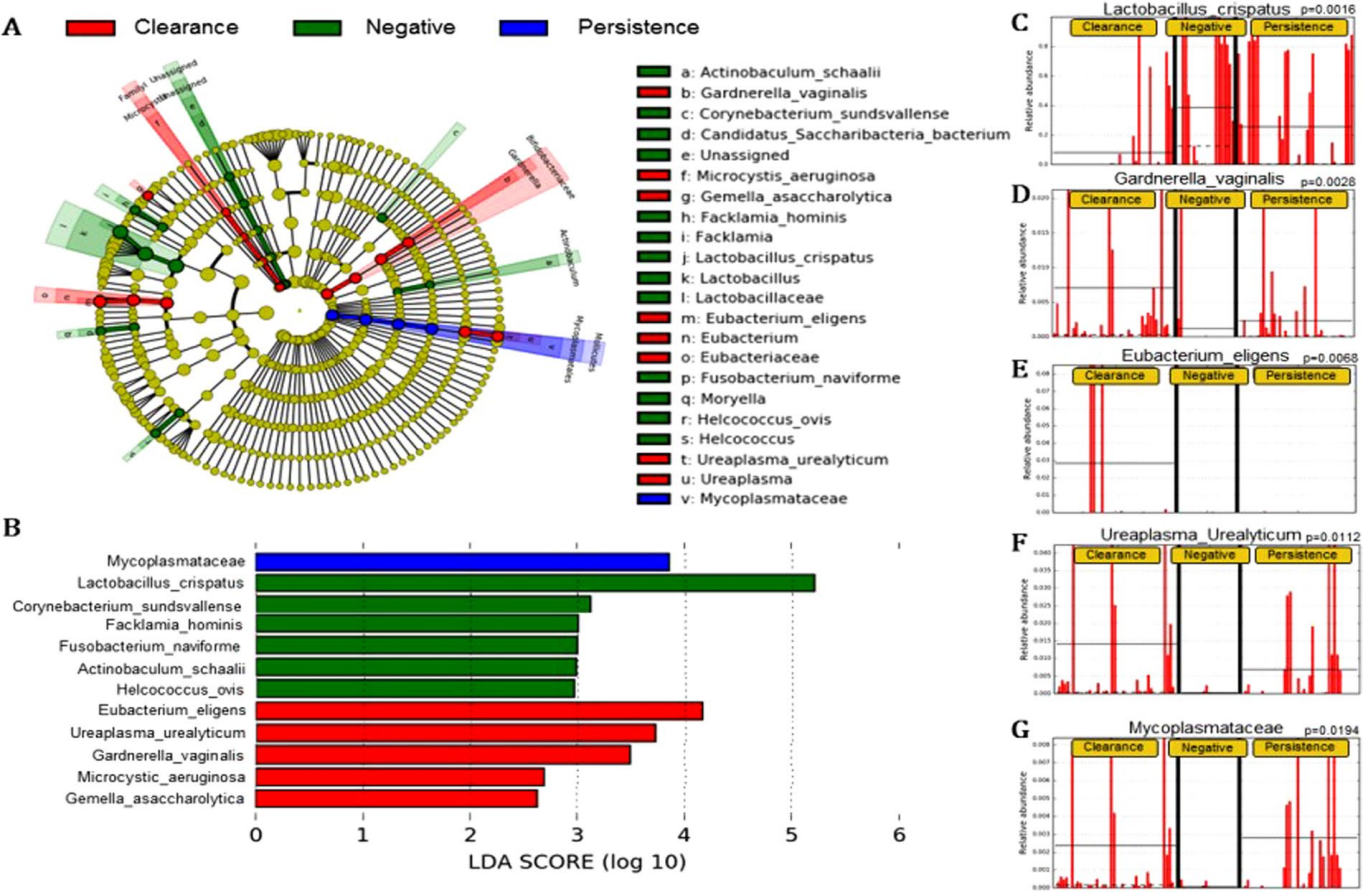
(**A**) Cladogram representing taxa with different abundances according to HPV groups in total visits. Red and green colors show taxa enriched in clearance and persistence women. Brightness is proportional to the abundance of taxa. (**B**) Histogram of Linear Discriminant Analysis (LDA) scores computed for features differentially abundant among HPV groups (clearance, negative and persistence). The LDA score on the log_10_ scale is indicated at the bottom. The greater the LDA score is, the more significant the phylotype biomarker is in the comparison. The relative abundance counts of *Lactobacillus crispatus*, *Gardnerella vaginalis*, *Eubacterium eligens*, *Ureaplasma urealyticum* and Mycoplasmataceae were found to be over-represented in the HPV negative (**C**), HPV clearance (**D**–**F**) and HPV persistence (**G**) groups.

The total-visit changes also are depicted in a cladogram (Fig. 2A). In the HPV-persistence group, the LDA score was highest for Mycoplasmataceae, which showed significant over-representation (P = 0.0194) (Fig. 2A,B,G). In the HPV-negative group, the highest LDA score was observed for *Lactobacillus crispatus*, followed by *Corynebacterium sundsvallense*, *Facklamia hominis*, *Fusobacteium naviforme*, *Antinobaculum schaali*, and *Helcococus ovis*, with significant over-representation of *Lactobacillus crispatus* (P = 0.0016) (Fig. 2A–C). In the HPV-clearance group, *Eubacterium eligens*, *Ureaplasma urealyticum*, and *Gardnerella vaginalis* were over-represented (Fig. 2A,B). The HPV-clearance group also was found to have significantly higher levels of *Gardnerella vaginalis* (P = 0.0028), *Eubacterium eligens* (P = 0.0068) and *Ureaplasma urealyticum* (P = 0.0112) (Fig. 2D–F).

**Figure 3:**
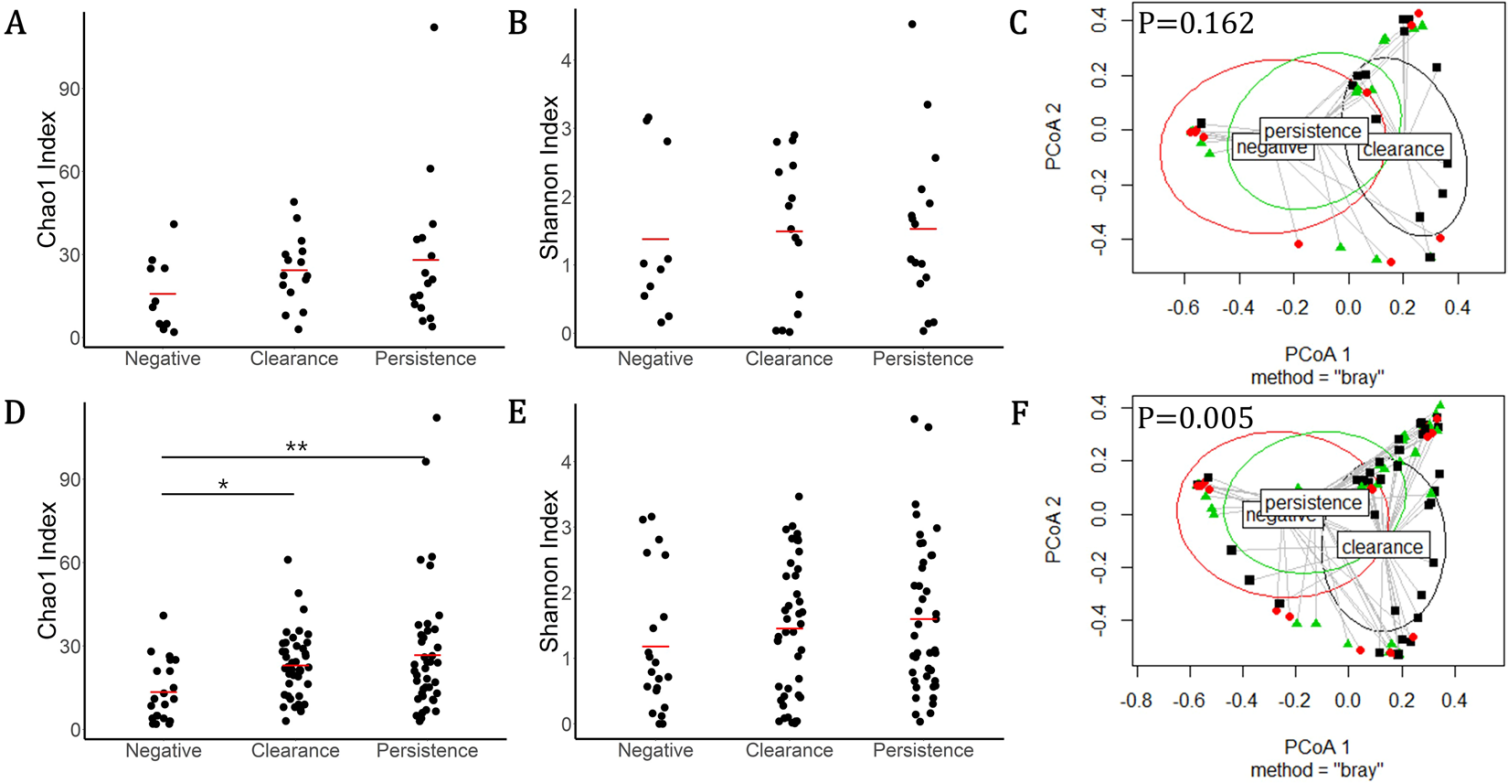
Chao1, alpha diversity (Shannon) and beta diversity index for three groups of women with baseline (**A**–**C**) and total-visit (**C**–**E**) samples. Values expressed in means. Statistical differences are represented by asteristics (<0.01) according to Kruskal-wallis test. ^*^p < 0.05, ^**^p < 0.01, ^***^p < 0.001.

We evaluated species richness (Chao 1 index), alpha-diversity (Shannon index) and beta-diversity of the cervical microbiome in baseline (Fig. 3A–C) and total visit samples (Fig. 3D–F). The results showed species richness (Chao1) was greater in the HPV-clearance and persistence subjects than in the HPV-negative subjects (p < 0.01) in total visits. Further, beta diversity differed between HPV negative, clearance and persistence group in total visit samples (bray p < 0.005). The baseline visit not showed any significance.

### Association of microbes with HPV clearance and HPV persistence

There were significant median differences in the relative abundances of *Lactobacillus crispatus*, *Eubacterium eligen*, *Ureaplasma urealyticum*, *Gardnerella vaginalis* and *Lactobacillus johnsonii* among the HPV groups. A multivariate logistic analysis (Table 2) showed that *Lactobacillus crispatus* (multivariate OR (mOR) = 8.25, 95% CI 2.13~32.0) was the most highly associated with HPV negativity. The multivariate OR of *Eubacterium eligen* for HPV clearance was higher (mOR = 11.5, 95% CI 1.31~101.4) than for HPV negativity. *Ureaplasma urealyticum* (mOR = 7.42, 95% CI 1.3~ 42.5) and *Gardnerella vaginalis* (mOR = 17.0, 95% CI 2.18–131.8) were more highly associated with HPV clearance than with HPV negativity. *Lactobacillus johnsonii* (mOR = 16.4, 95% CI 1.77~152.2) was associated more with HPV persistence than with HPV clearance.

**Table 2:** Multivariate odds ratios of species (relative abundance >0) f or HPV dynamics according the relative abundance of four species.

Group	N (%) (no exist/exist)^a^	Median (Relative abundance)	*p* ^b^	Multivariate OR (95% CI)^c^
*Lactobacillus crispatus*				
Clearance + Persistence (ref)	48 (55.8)/38 (44.2)	16.41		1 (ref)
Negative	5 (23.8)/16 (76.2)	38.39	0.009	8.25 (2.13~32.0)
*Eubacterium eligen*				
Negative + Persistence (ref)	61 (96.8)/2 (3.2)	0.009		1 (ref)
Clearance	34 (77.3)/10 (22.7)	12.32	0.001	11.5 (1.31~101.4)
*Ureaplasma urealyticum*				
Negative (ref)	18 (85.7)/3 (14.3)	0.015		1 (ref)
Clearance	21 (47.7)/23 (52.3)	1.253	0.003	7.42 (1.30~42.5)
*Gardnerella vaginalis*				
Negative (ref)	19 (90.5)/2 (9.5)	0	0.0002	1 (ref)
Clearance	18 (40.9)/26 (59.1)	0.03		17.0 (2.18–131.8)
*Lactobacillus johnsonii*				
Clearance (ref)	42 (95.5)/2 (4.5)	0	0.0101	1 (ref)
Persistence	33 (78.6)/9 (21.4)	0		16.4 (1.77–152.2)

^a^The number of women with/without each microbiome is presented.

^b^*p* value was from Wilcoxon rank-sum test and p < 0.05 was regarded as significant.

^c^Multivariate odds ratios and 95% CI of all variables were estimated by no exist (relative abundance = 0) as reference. All variables in this table are adjusted for age, menopausal status, oral contraceptive use and smoking habit as categorical types.

## Discussion

In the present study, we assessed the longitudinal associations of cervical microbes in HPV-negativity, clearance and persistence women. The main findings showed that the cervical microbiome differs significantly by HPV group. We found that women with high proportions of *Lactobacillus johnsonii*, Haemophilus (genus) and Mycoplasmataceae (family) had the strongest association with HPV persistence, and that women with a cervical microbiome dominated by *Eubacterium eligens*, *Gardnerella vaginalis* and *Ureaplasma urealyticum* had the strongest associations with HPV clearance. Women with a high proportion of *Lactobacillus crispatus* were most likely to have HPV-negative infection. Higher bacterial diversity was observed in HPV-persistence women than in women showing HPV negativity.

Based on these results of our two-year longitudinal study, we can suggest that bacterial dysbiosis is a factor associated with HPV dynamics (progression and regression). This study is, to the best of our knowledge, the first to examine the relationship between Haemophilus and *Lactobacillus johnsonii* in HPV-persistent women.

Our multivariate logistic analysis confirmed that *Lactobacillus johnsonii* was significantly associated (mORs = 16.4, 95% CI = 1.77–152.2) with HPV persistence among our Korean subjects. There is no previous study on *Lactobacillus johnsonii* in relation to cervical microbiomes, though it has been reported in saliva samples of HPV-positive and HPV-negative oropharyngeal cancer patients. A recent study by Guerrero-Preston *et al*. reported bacterial species *Lactobacillus gasseri/johnsonii* and *Lactobacillus vaginalis* in saliva of HPV-positive and HPV-negative oropharyngeal cancer patients^15^. However, the interaction between these microbes in saliva with HPV persistence and cancer risk was not discussed. The LEfSe analysis in the present study also confirmed enrichment of the genus Haemophilus and family Mycoplasmataceae in HPV-persistence women. The results for Mycoplasmataceae are in agreement with the finding of Abedamowo *et al*., that *Mycoplasma hominis* in cervical microbiota is significantly associated with hr-HPV infection^16^. It has also been reported that a few Mycoplasma species are efficient methylators and can promote cervical carcinogenesis through methylation of hr-HPV and cervical somatic cells^16^. One of the previous cervical microbiota studies (16-weeks samples) conducted by Brotman *et al*.^12^ demonstrated that high proportions of Atopobium spp. (belonging to CST IV-B) were associated with the slowest HPV clearance rate and that the persistence effect may be due to the high proportion of *Atophobium vaginae* that can play a role in the disruption of the epithelial barrier. The plausible molecular mechanisms of HPV persistence infection have been proposed: the viral life cycle and immune system evasion (avoidance of triggering of an immune response by the host)^17^, the tethering mechanism on host mitotic chromosomes (to ensure that the viral genome is not lost during cell division)^17^, and E2 interaction with host receptors^17^. Then, 16 S rRNA gene sequencing revealed an increased proportion of *L*. *johnsonii* in HPV persistence and depletion of *Lactobacillus crispatus* relative to HPV-negative women; additionally, an alpha-diversity analysis provided further confirmation that HPV persistence in the total-visit samples showed increased species richness. Therefore, it can be suggested that changes in cervical microbiota with depletion in *Lactobacillus crispatus* and increased microbial diversity promote HPV infection and might be involved in HPV persistence^18^. The alpha-diversity results (Chao 1 index) showed increased diversity in HPV-persistence women relative to HPV-negative women in the total-visit samples. Therefore, high bacterial diversity in HPV persistence can represent an environment that increases the risk of progressing HPV persistence.

In the HPV-clearance women, multivariate logistic analysis and LEfSe analysis confirmed the significant enrichment of species *G*. *vaginalis*, *E*. *eligens* and *Ureaplasma urealyticum*. The clearance effect might be due to the innate immune response (Toll-like receoptors-TLR) that is involved in a cascade of events promoting HPV clearance. TLR 3 and TLR9 play a role in regulating the pro-inflammatory cytokine and anti-viral environments of the lower female genital tract during viral and bacterial infection^19^; intense inflammatory response induced by *G*. *vaginalis* in viral clearance^20^, as well as viral proteins^21^ and other intrinsic host factors (such as sexual behaviors)^21,22^ responsible for HPV clearance. Another plausible explanation is that this clearance effect is due to an intense inflammatory response or that higher concentrations of inflammation markers (IP-10 and MIG chemokines) induced by bacteria in the cervical region assist in viral clearances^23^. One study conducted by Moscicki *et al*.^20^ reported that the HPV viral clearance effect might be due to the serendipity effect of *Neisseria gonorrhe*. One previous study^24^ reported the lowest clearance rate in women whose cervical microbiota were dominated by Atopobium and Gardnerella; therefore, further studies are needed to identify the mechanism or mechanisms whereby the composition of the cervical microbiota influence clearance.

Among the HPV-negative women, the *Lactobacillus crispatus* population remained stable at the baseline and follow-up visits, and *Lactobacillus crispatus* was significantly over-represented. Interestingly, in the LEfSe analysis results, we observed *Corynebacterium sundsvallense*, *Facklamia hominis*, *Actinobaculum schaalii* and *Helcococcus ovis* for the first time in the HPV-negative women. As reported earlier, in healthy women, hydrogen-peroxide producing Lactobacillus spp. are stable in maintaining the vaginal ecosystem^25^. Klebanoff *et al*. reported that Lactobacillus sp. increase TNF-α and IL-1α production, activate NF-κB in THP-1 cells and increase TNF-α production by human monocytes. This suggests that a higher concentration of Lactobacillus in the vagina can influence the physiology and host defenses therein^25^. In our previous study, we reported that *Lactobacillus crispatus* and *Lactobacillus inners* (Cluster III) were the dominant species in HPV-negative women and that high abundance of *L*. *crispatus* is associated with a low risk of CIN^26^.

The strength of our present research is that the HPV samplings were longitudinal and that the differences between the cervical microbial compositions (species and genus level) among the HPV-negativity, clearance and persistence samples were identified by 16 S rRNA pyrosequencing. One of the most notable findings is that the bacterial communities in the cervical region of the HPV-persistence and clearance women were distinctive from those in the HPV-negative women, suggesting the presence of certain selective pressures contributing to the shift in the cervical microbiota. However, we also must acknowledge certain study limitations: (1) small sample sizes among the study groups, which could have biased the results; (2) an advanced-age study population (mean age: 44). So, the results should be interpreted with caution, and especially, it should be recognized that they might not apply to the general population; (3) another minor limitation of this study is HPV grouping by the liquid hybridization assay Hybrid Capture 2 (HC2) assays. It has been noted that HC2 assay shows some false positive results of 5%. Despite these limitations, HC2 assay has several advantages over other commercially available PCR-based target amplification method^27–29^. Moreover, HC2 assay is technically well designed and can be easily controlled and performed by the laboratory technician. Whereas PCR-based method contains several steps that must be carefully optimized which make it more difficult to standardize PCR in laboratory condition.

In conclusion, this study analyzed various cervical microbial taxa among different HPV groups, and found that increased bacterial diversity with reduced *L*. *crispatus* species could be related to HPV persistence and also that the presence and prevalence of a specific cervical microbiome could be relevant to HPV dynamics.

## Methods

### Subject recruitment

We collected cervical samples from Normal and ASCUS patients (age between 18 and 65 years) who had participated in the Korean human papillomavirus (HPV) cohort study from 2006 to 2013. Detailed information on the inclusion/exclusion criteria for the HPV cohort is provided in our previous paper^30^. The selected participants were interviewed using a structured questionnaire on their socio-demographic characteristics (Table 1), after which they underwent physical and gynecological examinations. Cervical samples were collected using a Cervix brush (Rovers Medical Devices, Oss, The Netherlands), after which the brush was immediately soaked in a vial of PreservCyt solution (Cytyc Corporation, Marlborough, MA, USA) fixed within a Thin Prep processor. The cytological findings were grouped based on the Bethesda system^31^. The samples were examined for the presence of one or more hrHPV types using the Hybrid Capture 2 (HC2) assay (16, 18, 31, 33, 35, 39, 45, 51, 52, 56, 58, 59, and 68) according to the manufacturer’s instructions. The values obtained were recorded in relative light units (RLUs). All relative light units (RLU) measured on a luminometer were divided by the RLU of the respective positive control to provide a ratio. The sample was classified as a positive when the RLU/PC ratio was 1 pg/mL or greater. After baseline sampling, follow-up visits were scheduled at six-month intervals. The collected samples were stored at −80 °C for further analysis.

### Grouping of HPV status for 2-year follow-up

**Figure 4:**
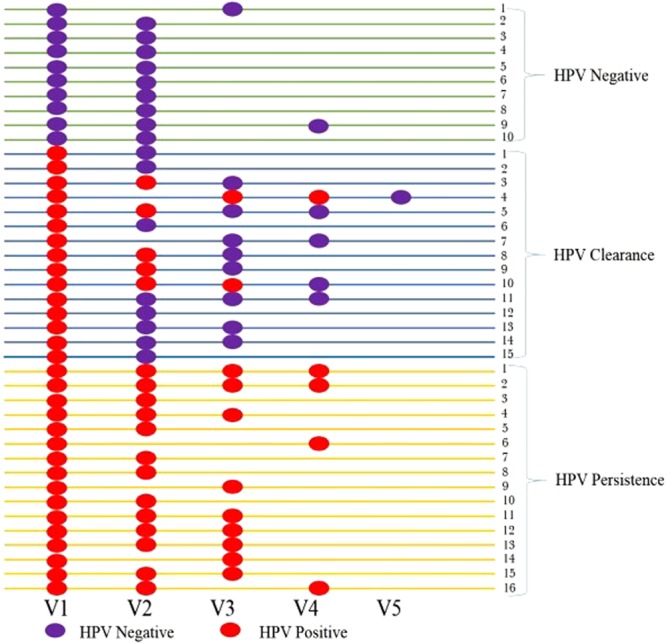
Grouping of HPV status for 2-year follow-up study. V1: baseline visit, V2: visit after 6 months, V3: visit after 12 months, V4: visit after 18 months, V5: visit after 24 months. Only available variables were used in this study, due to missing responses for several questions.

To investigate the shifts of bacterial communities in the HPV states, we grouped the samples based on the following conditions: (1) HPV negative – persistently negative detection of hrHPV types throughout 24 months (all negative during observation period, 21 samples, 10 subjects); (2) HPV clearance – women who were initially hrHPV (baseline), and then regressed to negative during follow-up (42 samples, 15 subjects); (3) HPV persistence – persistently positive detection of hrHPV types at the baseline and during follow-up (all positive during observation period, 44 samples, 16 subjects) (Fig. 4). Of the 41 subjects, 35 participated at the 2^nd^ visit, 20 at the 3^rd^ visit, 10 at the 4^th^ visit, and 1 at the final visit.

### High-risk HPV DNA detection

HPV DNA was detected using the Digene HC2 high-risk DNA test (Qiagen, Gaithersburg, MD, USA) with signal amplification and chemiluminescence for 13 types of HR-HPV scored in RLU/PC. A positive result indicated a concentration of 1 pg/ml or higher than the RLU/cutoff ratio (RLU of the specimen/mean RLU of 2 positive controls).

### DNA isolation and pyrosequencing

DNA from cervical samples were isolated using the Fast DNA SPIN kit (MP Biomedicals, Santa Ana, CA, USA). The 16 S universal primers 27 F (5′ GAGTTTGATCMTGGCTCAG 3′) and 518 R (5′ WTTACCGCGGCTGC-TGG 3′) were used for amplification. A 20 ng aliquot of each sample was used for a 50 µl PCR reaction containing 10 × taq buffer, a dNTP mixture (Takara, shiga, Japan), 10 μm of the bar-coded fusion primers, and 2 U of taq Polymerase (extaq, takara). The PCR conditions and pyrosequencing protocols are available elsewhere^32^. Beads recovered from emulsion PCR were deposited on a 454 Pico titer plate, and sequencing was executed using a Roche/454 GS-FLX plus. The sequencing run was performed by Macrogen Ltd. (Seoul, Korea).

### Next-Generation Sequencing using 454 GS-FLX plus

The raw sequences for the samples were arranged using a unique barcode, and low-quality reads (average quality score <25 or read length <300 bp) were removed^32^. The primer sequences were cut down by employing pairwise sequence alignment, and sequences were gathered to correct for sequencing errors. Taxonomic identification was performed using the EzTaxon-e public database^33^ according to the highest pairwise similarity among the BLASTN search results. Possible chimera sequences were removed by the UCHIME algorithm^34^, and the diversity indices were calculated in Mothur after normalization of the read number in each sample. The potential biomarkers linked to HPV negative, HPV clearance and HPV persistence were analyzed by LDA effect size (LEfSe). Finally, the effect relevance was predicted by LDA^35^.

### Statistical analysis

The Chi-square test was used for testing of the categorical variables, and the Kruskal Wallis test was employed for comparison of the categorical continuous variables. When the number of expected frequencies was less than 5 and the number of cells was more than 25%, Fisher’s exact test was performed. To find a significant difference in alpha diversity, the Kruskal Wallis rank sum test was used to evaluate the differences in diversity among the three groups, followed by Dunn’s test of multiple comparisons. Multivariate logistic analysis was performed after adjusting for age, menopausal status, oral contraceptive use, and smoking habit. Risk estimates are presented as OR with 95% CI. LEfSe analysis was conducted to find significant differences among the relative abundance taxa^35^. Beta diversity was calculated with principal coordinates analysis (PCoA) according to the Bray-Curtis distances. A permutational multivariate analysis of variance (PERMANOVA) was implemented to determine significance in distance. Diversity and the PERMANOVA results were analyzed using the R packages “vegan“^36^. The statistical analysis was performed with SAS 9.4, and R version 3.3.1 with ggplot2 packages was used for visualization^37^.

### Ethical Statement

The Ethics Committee of the National Cancer Center approved this study, and all of the experiments were performed in accordance with the approved guidelines and regulations (IRB No. NCC2016-0147). Written informed consent was obtained from all of the study participants in accordance with good clinical practice.

## Electronic supplementary material

Supplementary Table S1

## Data Availability

The datasets generated during the current study are available from corresponding author on reasonable request.

## References

[1] Bosch FX, Lorincz A, Munoz N, Meijer CJ, Shah KV (2002). The causal relation between human papillomavirus and cervical cancer. J Clin Path.

[2] Munoz N, Castellsague X, de Gonzalez AB, Gissmann L (2006). Chapter 1: HPV in the etiology of human cancer. Vaccine.

[3] Ho GY, Bierman R, Beardsley L, Chang CJ, Burk RD (1998). Natural history of cervicovaginal papillomavirus infection in young women. The N Engl J Med.

[4] Schettino MT (2014). Persistent papillomavirus type-31 and type-45 infections predict the progression to squamous intraepithelial lesion. Taiwan J Obst Gyn.

[5] Walboomers, J. M. *et al*. Human papillomavirus is a necessary cause of invasive cervical cancer worldwide. The *J Pat* **189**, 12–19, 10.1002/(sici)1096-9896(199909)189:1<12::aid-path431>3.0.co;2-f (1999). 10.1002/(SICI)1096-9896(199909)189:1<12::AID-PATH431>3.0.CO;2-F 10451482

[6] Castellsague X., Munoz N. (2003). Chapter 3: Cofactors in Human Papillomavirus Carcinogenesis--Role of Parity, Oral Contraceptives, and Tobacco Smoking. JNCI Monographs.

[7] Dahlstrom LA (2011). Prospective seroepidemiologic study of human papillomavirus and other risk factors in cervical cancer. Can Epi Bio Prev..

[8] Mitra A (2016). The vaginal microbiota, human papillomavirus infection and cervical intraepithelial neoplasia: what do we know and where are we going next?. Microbiome.

[9] Song D, Li H, Li H, Dai J (2015). Effect of human papillomavirus infection on the immune system and its role in the course of cervical cancer. Onc Let.

[10] Kjaer SK (2005). Acquisition and persistence of Human Papillomavirus Infection in Younger Men: A Prospective Follow-up Study among Danish Soldiers. Can Epi Bio Prev.

[11] Goodman MT (2008). Prevalence, acquisition, and clearance of cervical human papillomavirus infection among women with normal cytology: Hawaii Human Papillomavirus Cohort Study. Can Res.

[12] Brotman RM (2014). Interplay between the temporal dynamics of the vaginal microbiota and human papillomavirus detection. The J of Inf Dis.

[13] Srinivasan S (2010). Temporal Variability of Human Vaginal Bacteria and Relationship with Bacterial Vaginosis. PloS One.

[14] Gajer P (2012). Temporal dynamics of the human vaginal microbiota. Sci Tran Med.

[15] Preston RG (2017). High-resolution microbiome profiling uncovers Fusobacterium nucleatum, Lactobacillus gasseri/johnsonii, and Lactobacillus vaginalis associated to oral and oropharyngeal cancer in saliva from HPV positive and HPV negative patients treated with surgery and chemo-radiation. Oncotarget.

[16] Adebamowo SN (2017). Mycoplasma hominis and Mycoplasma genitalium in the Vaginal Microbiota and Persistent High-Risk Human PapillomavirusInfection. Front in Pub Health.

[17] Shanmugasundaram, S. S. & You, J. Targeting Persistent Human Papillomavirus Infection. *Viruses* **9**10.3390/v9080229 (2017). 10.3390/v9080229 PMC5580486 28820433

[18] Paola MD (2017). Characterization of cervico-vaginal microbiota in women developing persistent high-risk Human Papillomavirus infection. Sci Rep.

[19] Andersen JM, Khairy AA, Ingalls RR (2006). Innate Immunity at the Mucosal Surface: Role of Toll-Like Receptor 3 and Toll-Like Receptor 9 in Cervical Epithelial Cell Responses to MicrobialPathogens. Bio Reprod.

[20] Moscicki AB (2010). Rate of and Risks for Regression of CIN-2 in adolescents and young women. Obst & Gynecol.

[21] Bhat P, Mattarollo SR, Gosmann C, Frazer IH, Leggatt GR (2011). Regulation of immune responses to HPV infection and during HPV-directed immunotherapy. Immunol Rev.

[22] Schmeink CE (2013). Prospective follow-up of 2,065 young unscreened women to study human papillomavirus incidence and clearance. Int J of Cancer.

[23] Shannon B (2017). Association of HPV infection and clearance with cervicovaginal immunology and the vaginal microbiota. Mucosal Immunol.

[24] Ledger J.W. W. S. S. *Chapter 9*. *Human Papillomavirus gential inflections*. *Second Edition*. 93 (2016).

[25] Klebanoff SJ, Hillier SL, Eschenbach DA, Waltersdorph AM (1991). Control of the microbial flora of the vagina by H2O2-generating Lactobacilli. J Infect Dis.

[26] Oh HY (2015). The association of uterine cervical microbiota with an increased risk for cervical intraepithelial neoplasia in Korea. Clinical Microbiol Infect.

[27] Castle PE (2008). Human papillomavirus genotype specificity of hybrid capture 2. J Clin Microbiol.

[28] Kurian EM (2011). Cervista HR and HPV 16/18 assays vs hybrid capture 2 assay: outcome comparison in women with negative cervical cytology. Am J Clin Pathol.

[29] Zhao C, Yang H (2012). Approved assays for detecting HPV DNA—design, indications, and validation. CAP Today.

[30] Hwang Jong Ha, Lee Jae Kwan, Kim Tae Jin, Kim Mi Kyung (2009). The association between fruit and vegetable consumption and HPV viral load in high-risk HPV-positive women with cervical intraepithelial neoplasia. Cancer Causes & Control.

[31] Solomon D (2002). The 2001 Bethesda System: terminology for reporting results of cervical cytology. JAMA.

[32] Jeon YS, Chun J, Kim BS (2013). Identification of household bacterial community and analysis of species shared with human microbiome. Curr Microbiol.

[33] Kim OS (2012). Introducing EzTaxon-e: a prokaryotic 16S rRNA gene sequence database with phylotypes that represent uncultured species. Int J Syst Evol Microbiol.

[34] Edgar RC, Haas BJ, Clemente JC, Quince C, Knight R (2011). UCHIME improves sensitivity and speed of chimera detection. Bioinformatics.

[35] Segata N (2011). Metagenomic biomarker discovery and explanation. Genome Biol.

[36] Oksanen, J. *et al*. Vegan: community ecology package, version 2.4–1, https://cran.r-project.org/web/packages/vegan/index.html

[37] Wickham, H. *ggplot2: elegant graphics for data analysis*. (Springer, 2016).

