# Supplementary material for: Association of cervical microbial community with persistence, clearance and negativity of Human Papillomavirus in Korean women: a longitudinal study
Source: Sci Rep. 2018 Oct 19;8:15479. doi: 10.1038/s41598-018-33750-y (PMC6195586; doi:10.1038/s41598-018-33750-y)
Supplement: Supplementary file 1 — Supplementary Table S1 [file 41598_2018_33750_MOESM1_ESM.docx]

**Association of cervical microbial community with persistence, clearance and negativity of Human Papillomavirus in Korean women: a longitudinal study**

Selvaraj Arokiyaraj^1,2†^, Sang Soo Seo^1†^, Minji Kwon^1^, Jae Kwan Lee^3^, Mi Kyung Kim^1^*

^1^Division of Cancer Epidemiology and Management, Center for Uterine Cancer, National Cancer Center, Ilsandong-gu, Goyang, Republic of Korea

^2^Department of Food Science and Biotechnology, Sejong University, Gwanjin-gu, Republic of Korea

^3^Department of Obstetrics and Gynecology, Korea University College of Medicine, Seoul, Republic of Korea

^†^ These authors contributed equally.

*Corresponding author: Mi Kyung Kim

Division of Cancer Epidemiology and Prevention, National Cancer Center, 323, Ilsan-ro, Ilsandong-gu, Goyang-si, Gyeonggi-do 411-769, Republic of Korea

E-mail: alrud@ncc.re.kr, Tel: +82-31-920-2202, Fax: +82-31-920-2006

**Supplementary Table S1 Distribution of cervical microbiota species at different time period among HPV negative, clearance and persistence women**

| **Bacteria** | **HPV negative** | | | |  | **HPV clearance** | | | | |  | | **HPV persistence** | | | | |
| --- | --- | --- | --- | --- | --- | --- | --- | --- | --- | --- | --- | --- | --- | --- | --- | --- | --- |
|  | **V1** | **V2** | **V3** | **V4** |  | **V1** | **V2** | **V3** | **V4,5** |  | | **V1** | | **V2** | **V3** | **V4** |  |
| ***Mobiluncus mulieris*** | 0.01 | 0 | 0 | 0 |  | 0.39 | 0 | 1.59 | 0.07 |  | | 0 | | 3.75 | 1.75 | 0.1 |  |
| ***Varibaculum cambriense*** | 1.01 | 0.03 | 0 | 0 |  | 0 | 0 | 0 | 0 |  | | 0 | | 0.04 | 0 | 0 |  |
| ***Gardnerella vaginalis*** | 0.03 | 0 | 2.33 | 0 |  | 0.74 | 1.25 | 0.09 | 0.06 |  | | 0.24 | | 0.35 | 0.14 | 0.01 |  |
| ***Atopobium vaginae*** | 2.4 | 3.46 | 33.76 | 0 |  | 9.36 | 7.58 | 7.91 | 0.99 |  | | 9.65 | | 8.49 | 10.62 | 9.37 |  |
| ***Eggerthella sp.*** | 0.4 | 0.09 | 0 | 0 |  | 0.91 | 1.62 | 1.1 | 1.46 |  | | 0.78 | | 0.78 | 2.44 | 1.66 |  |
| ***Prevotella amnii*** | 2.17 | 0.04 | 0 | 0 |  | 1.67 | 0.52 | 3.77 | 4.52 |  | | 0 | | 1.82 | 0.56 | 3.76 |  |
| ***Prevotella bivia*** | 0.07 | 0 | 18.73 | 0.19 |  | 3.75 | 1.69 | 2.28 | 0 |  | | 3.58 | | 1.14 | 1.75 | 0.15 |  |
| ***Prevotella buccalis*** | 5.72 | 8.2 | 0 | 0 |  | 3.17 | 2.3 | 1.94 | 2.63 |  | | 4.44 | | 10.42 | 6.48 | 4.94 |  |
| ***Prevotella disiens*** | 0 | 0.01 | 0 | 0 |  | 0 | 0.07 | 0 | 0 |  | | 0.04 | | 0.04 | 1.06 | 0 |  |
| ***Staphylococcus haemolyticus*** | 0.02 | 0 | 0.04 | 0 |  | 0 | 0.16 | 0 | 3.04 |  | | 0 | | 0 | 0.01 | 0.74 |  |
| ***Aerococcus christensenii*** | 0.01 | 0.16 | 9.09 | 0 |  | 3.29 | 1.65 | 0.09 | 0 |  | | 2.29 | | 0.07 | 4.68 | 0.12 |  |
| ***Lactobacillus crispatus*** | 40.45 | 35.6 | 0 | 81.31 |  | 5.16 | 5.67 | 4.72 | 25.76 |  | | 23.67 | | 25.15 | 18.12 | 47.39 |  |
| ***Lactobacillus fornicalis*** | 5.12 | 3.18 | 0 | 18.51 |  | 0.97 | 6.7 | 1.34 | 3.55 |  | | 5.03 | | 12.97 | 3.55 | 6.73 |  |
| ***Lactobacillus iners*** | 23.27 | 28.28 | 3.6 | 0 |  | 31.57 | 34.66 | 24.8 | 34.26 |  | | 16.39 | | 13.93 | 22.26 | 1.71 |  |
| ***Lactobacillus johnsonii*** | 0.01 | 0.03 | 0 | 0 |  | 0 | 0 | 0.05 | 0 |  | | 6.15 | | 7.02 | 0.16 | 0 |  |
| ***Streptococcus agalactiae*** | 4.55 | 10.1 | 0 | 0 |  | 6.07 | 7.59 | 9.48 | 3.17 |  | | 4.04 | | 0.3 | 2.31 | 2.1 |  |
| ***Streptococcus anginosus*** | 0.26 | 0.12 | 0 | 0 |  | 0.63 | 7.59 | 6.85 | 0 |  | | 0.75 | | 0.17 | 6.18 | 3.93 |  |
| ***Streptococcus rubneri*** | 0.19 | 0.01 | 0 | 0 |  | 0.01 | 0 | 0.09 | 0 |  | | 2.45 | | 0.23 | 0 | 0.31 |  |
| ***Streptococcus salivarius*** | 0.01 | 0 | 0 | 0 |  | 0 | 0.01 | 0 | 0 |  | | 3.87 | | 0 | 0 | 0 |  |
| ***Eubacterium eligens*** | 0 | 0 | 0 | 0 |  | 2.46 | 0 | 7.32 | 2.43 |  | | 0 | | 0.01 | 0 | 0 |  |
| ***Clostridiales genomosp_*** | 2.4 | 0.16 | 0 | 0 |  | 0.06 | 0.01 | 0.09 | 0.27 |  | | 0 | | 0.93 | 1.17 | 0.06 |  |
| ***Clostridium straminisolvens*** | 2.53 | 1.57 | 13.86 | 0 |  | 1.74 | 0.67 | 1.57 | 1.21 |  | | 1.3 | | 1.5 | 1.71 | 1.9 |  |
| ***Faecalitalea cylindroides*** | 0 | 0 | 0 | 0 |  | 0 | 0 | 0 | 0 |  | | 0 | | 2.42 | 0.01 | 0 |  |
| ***Dialister micraerophilus*** | 0.45 | 0.08 | 1.49 | 0 |  | 0.58 | 1.07 | 0.74 | 0.15 |  | | 0.52 | | 1.29 | 2.41 | 0.38 |  |
| ***Megasphaera micronuciformis*** | 1.7 | 0.48 | 16.66 | 0 |  | 4.98 | 6.05 | 3.7 | 3.57 |  | | 3.7 | | 2.17 | 1.86 | 0.57 |  |
| ***Sneathia sanguinegens*** | 2.35 | 6.3 | 0.18 | 0 |  | 11.47 | 3.02 | 15.62 | 3.55 |  | | 0.04 | | 2.22 | 2.8 | 2.95 |  |
| ***Escherichia coli*** | 0 | 0 | 0 | 0 |  | 4.65 | 0 | 0.08 | 5.39 |  | | 0.16 | | 0 | 0 | 0 |  |
| ***Pseudomonas fluorescens*** | 0 | 0 | 0 | 0 |  | 0.02 | 6.62 | 0 | 2.84 |  | | 0 | | 0.01 | 0.02 | 0 |  |
| ***Mycoplasma hominis*** | 0.03 | 0 | 0.04 | 0 |  | 0.01 | 0 | 0.04 | 0.02 |  | | 0.1 | | 0.08 | 4.32 | 0 |  |
| ***Ureaplasma urealyticum*** | 0.01 | 0.03 | 0 | 0 |  | 2.01 | 1.46 | 0.6 | 0.005 |  | | 1.1 | | 0.14 | 0.7 | 0.55 |  |
| ***Other*** | 0.04 | 0.01 | 0 | 0 |  | 2.87 | 0.73 | 0.6 | 0.07 |  | | 7.82 | | 0.57 | 0.92 | 5.86 |  |

Relative abundance of cervical microbial species >1% are shown in this table. V1: baseline visit, V2: 6 months, V3: 12 months, V4: 18 months, V5: 24 months.
